# A Tandem Repeat in Decay Accelerating Factor 1 Is Associated with Severity of Murine Mercury-Induced Autoimmunity

**DOI:** 10.1155/2014/260613

**Published:** 2014-04-10

**Authors:** David M. Cauvi, Rodney Gabriel, Dwight H. Kono, Per Hultman, K. Michael Pollard

**Affiliations:** ^1^Department of Surgery, University of California, San Diego, 9500 Gilman Drive, No. 0739, La Jolla, CA 92093-0739, USA; ^2^Department of Molecular and Experimental Medicine, The Scripps Research Institute, La Jolla, CA 92037, USA; ^3^Department of Immunology and Microbial Science, The Scripps Research Institute, La Jolla, CA 92037, USA; ^4^Molecular and Immunological Pathology, Department of Experimental and Clinical Medicine, Linköping University, 581 83 Linköping, Sweden

## Abstract

Decay accelerating factor (DAF), a complement-regulatory protein, protects cells from bystander complement-mediated lysis and negatively regulates T cells. Reduced expression of DAF occurs in several systemic autoimmune diseases including systemic lupus erythematosus, and DAF deficiency exacerbates disease in several autoimmune models, including murine mercury-induced autoimmunity (mHgIA). * Daf1*, located within * Hmr1*, a chromosome 1 locus associated in DBA/2 mice with resistance to mHgIA, could be a candidate. Here we show that reduced * Daf1* transcription in lupus-prone mice was not associated with a reduction in the * Daf1* transcription factor SP1. Studies of NZB mice congenic for the mHgIA-resistant DBA/2 * Hmr1* locus suggested that * Daf1* expression was controlled by the host genome and not the * Hmr1* locus. A unique pentanucleotide repeat variant in the second intron of * Daf1* in DBA/2 mice was identified and shown in F2 intercrosses to be associated with less severe disease; however, analysis of * Hmr1* congenics indicated that this most likely reflected the presence of autoimmunity-predisposing genetic variants within the *Hmr1* locus or that *Daf1* expression is mediated by the tandem repeat in epistasis with other genetic variants present in autoimmune-prone mice. These studies argue that the effect of DAF on autoimmunity is complex and may require multiple genetic elements.

## 1. Introduction

Decay accelerating factor (DAF [the gene and protein designations for decay accelerating factor in this paper are* DAF* for the human gene and DAF for the human protein; the mouse genes are* Daf1* and* Daf2* and corresponding proteins DAF1 and DAF2] or CD55) is a surface-expressed member of the complement-regulatory protein family that protects cells from attack by autologous complement proteins [1]. DAF inhibits the neoformation and accelerates the dissociation of preformed C3/C5 convertase complexes generated by the classical and alternative pathways, thus blocking both complement split product activity and the formation of the membrane attack complex [2]. DAF is present on inflammatory cells and at sites of tissue inflammation where it most likely inhibits bystander complement-mediated cell lysis (13). In addition, recent studies suggest DAF regulates T cell activity [3–6].

In humans, DAF is a single gene on chromosome 1q32 encoding a glycosylphosphatidylinositol- (GPI-) anchored cell surface glycoprotein [7]. In contrast, mice have two tandem Daf genes positioned head-to-tail on chromosome 1 [8] with the GPI-linked* Daf1* (Daf-GPI) located 5′ to the transmembrane containing* Daf2* (Daf-TM). Expression of DAF varies depending on tissue [9] and cell type [10] with DAF widely expressed on the surface of all major circulating blood cells and epithelial and endothelial cells [11, 12]. Studies with* Daf1* knockouts showed that absence of the GPI form results in the loss of DAF expression in most tissues, except testis and spleen where* Daf2* is expressed (27, 28). In the spleen, DAF2 is expressed primarily in CD11c^+^ dendritic cells (28). In human cells, DAF expression is modulated by cytokines such as IL-1, IL-6, TNF-*α*, TGF-*β*1, and IFN-*γ* [13–15], prostaglandin PGE2 [16], and tissue specific factors [17]. Although there is evidence that* DAF* mRNA stability can be affected by tissue specific factors [17] and inflammation [18], most studies suggest that expression is primarily modulated at transcription [15–17, 19, 20]. The human* DAF* promoter has been identified, the transcription start site mapped, and regions of potential transcriptional regulation proposed [10, 21]. Analysis of the key transcriptional regulatory elements controlling basal expression of mouse* Daf1* showed that transcriptional activity requires the functional cooperation of two Sp1-binding sites and is enhanced by the presence of a CREB site [22].

Evidence supports a protective role for DAF in autoimmunity [6, 23].* Daf1* deficient mice exhibit increased CD4^+^ T cell proliferation and greater secretion of IFN-*γ*, IL-2, and IL-4 but reduced IL-10 [4]. Furthermore, DAF1 is reduced on T and B cells in autoimmune prone NZB mice [24], and its deletion in lupus prone MRL-*Fas* ^*lpr*^ mice accelerates disease [25]. During induction of murine mercury-induced autoimmunity (mHgIA) DAF1 is specifically reduced on CD4^+^ T cells resulting in an accumulation of activated (CD44^high^Daf^low^) CD4^+^ T cells [24].* Daf1* deficiency also exacerbates mHgIA via increased levels of IFN-*γ*, IL-2, IL-4, and IL-10 but not IL-17 [26]. DAF mediated complement regulation does not appear to contribute to mHgIA as neither the accumulation of CD44^high^Daf^low^ CD4^+^ T cells nor the downregulation of DAF1 expression on CD4^+^ T cells was influenced by a lack of C3 [27]. Additionally,* Daf1* deficiency exacerbates organ specific disease in models of experimental autoimmune encephalomyelitis (EAE) [4], glomerulonephritis in antibody-induced nephritis [28, 29], and experimental myasthenia gravis [30]. Thus DAF impacts the expression of disease in both idiopathic and induced models of autoimmunity.

In a previous study, we showed that resistance to mHgIA resides at a single major quantitative trait locus on chromosome 1, designated* Hmr1*, which was shown to be linked to glomerular immune complex deposits but not autoantibody production [31].* Hmr1* encompasses a region containing several lupus susceptibility loci as well as* Daf1* and* Daf2*. As DAF regulates complement activation it is possible that differences in DAF expression may impact the deposition of immune complex deposits and contribute significantly to the* Hmr1* phenotype. In this study, we show that* Daf1* expression is reduced in multiple murine strains susceptible to spontaneous autoimmunity and identified a pentanucleotide tandem repeat in the second intron of* Daf1, *which in the mHgIA resistant DBA/2 consisted of eleven repeats while most other strains had 10, except for MRL-*Fas* ^*lpr*^ and SJL/J, which lacked the repeat. Comparison of the presence or absence of the tandem repeat, in a (DBA/2xSJL/J)F2 intercross, with several disease parameters showed that presence of the DBA/2 repeat was associated with less severe disease. Analysis of NZB mice congenic for the* Hmr1* locus of DBA/2, however, showed that* Daf1* expression is controlled by trans elements not within* Hmr1* and the reduction in* Daf1* expression was not associated with changes in levels of its major transcription factor SP1. These studies document lower levels of* Daf1* in lupus-prone mice and show that this is not directly caused by cis elements within the* Daf1* gene or by differences in constitutive Sp1 expression.

## 2. Materials and Methods

### 2.1. Mice

DBA/2, NZB, MRL/Fas^lpr^/J, BXSB, and C57BL/6 mice were obtained from the Scripps Research Institute Breeding Colony (La Jolla, CA). NZW/LacJ, A.SW/SnJ, BALB/cJ, SJL/J, and 129S6 mice were obtained from the Jackson Laboratory (Bar Harbor, ME). (SJL/JxDBA/2)F2 intercross mice have been previously described [31]. NZB.DBA/2-*Hmr1(Daf*1^*DBA*/2^) and DBA/2.NZB-*Hmr1(Daf*1^*NZB*^) interval congenic mice that contained the relevant* Daf1* locus were generated by marker-assisted breeding using* D1Mit21* (67 Mb) and* D1Mit17* (190 Mb) to define the outer limits of the chromosome 1 interval. Breeding and maintenance were performed under specific pathogen-free conditions at the Scripps Research Institute Animal Facility (La Jolla, CA). All procedures were approved by the Scripps Research Institute's Institutional Animal Care and Use Committee.

### 2.2. RNA Isolation and Real-Time PCR

Total RNA extraction from splenocytes was performed using TRIzol reagent (Invitrogen, Carlsbad, CA). RNA was denatured at 65°C for 5 minutes, placed on ice, and reverse transcribed in a total volume of 20 ml using random hexamers, dNTPs, RNase inhibitor (RNase-OUT; Invitrogen), and 200 units of SuperScript III reverse transcriptase (Invitrogen). Real time PCR primers, probes, and methods were as previously described [24].* Daf1* was expressed relative to cyclophilin A or 18sRNA [24]. Levels of Sp1, Sp3, CREB, and CREM mRNA were determined in spleen cells of naïve female autoimmune prone NZB and healthy DBA/2 mice by real time PCR using iQ SYBR green Supermix (Bio-rad, Hercules, CA). The following primers were used for amplification: Sp1 forward, 5′-CAAACACCCCAGGTGATCATGGAAC-3′, and Sp1 reverse, 5′-CAGTGAGGGAAGAGCCTCAGGAG-3′; Sp3 forward, 5′-GGCAGCTCAGTGGTGACTCTAC-3′, and Sp3 reverse, 5′GGTGGTGGGAGAGGTACCAATC-3′; CREB forward, 5′-GTGGGCAGTACATTGCCATTACCC-3′, and CREB reverse, 5′-GTTGTTCAAGCTGCCTCAGGCG-3′; CREM forward, 5′-CACAGGTGACATGCCAACTTACCAG-3′, and CREM reverse, 5′-CGGGAGTGTCGCAGGAAGAAG-3′. All PCR reactions were performed using an iCycler iQ (Bio-Rad). The reactions were run in duplicate and relative expression mRNA levels were determined by the ΔΔCT method and normalized against cyclophilin A. Data are expressed as fold change compared to mRNA levels measured in DBA/2 samples.

### 2.3. Genomic DNA

Genomic DNA was isolated from 5 mm sections of mouse tail incubated in 500 *μ*L lysis buffer (0.1 M Tris, pH 8.0; 5 mM EDTA; 0.2 M NaCl and 0.4% w/v SDS) containing 200 *μ*g/mL proteinase K (Sigma, St Louis, MO) overnight at 55°C. Samples were spun down and supernatants containing genomic DNA were purified using the ZR Genomic DNA^TM^-Tissue MiniPrep kit (Zymo Research, Irvine, CA) according to the manufacturer's protocol.

### 2.4. Tandem Repeat PCR

The tandem repeat sequence, which can be represented by either (CTTTT)n or (TTTTC)n, was identified within the second intron of Daf1 using the following primers: 5′-GCTTAAGGCATTACTGTCTGC-3′ (forward) and 5′-GCCATCCTAATGTAAAGTAACTCC-3′ (reverse). PCR amplifications were performed with the KOD Hot Start Polymerase (EMD Millipore, Billerica, MA) using the following conditions: an initial 2 min denaturation step at 94°C and then 35 cycles of denaturation (94°C), annealing (57°C), and extension (68°C) followed by a final 10 min 68°C incubation step. The PCR products were separated by agarose gel electrophoresis, extracted using the QIAquick Gel Extraction Kit (Qiagen, Valencia, CA), and submitted for sequencing.

### 2.5. DNA Sequencing

Purified PCR products were submitted to the Scripps Research Institute DNA Core Facility and analyzed with an ABI PRISM 3100 sequencer using appropriate primers. Sequencing data were analyzed with the BioEdit Sequence Alignment Editor software.

### 2.6. Induction and Assessment of mHgIA in (SJL/JxDBA/2)F2 Intercross Mice

Induction and features of mHgIA including immune deposits in kidney and spleen and serum autoantibodies and MHC class II genotypes in (SJL/JxDBA/2)F2 intercross mice were described previously [31]. Use of mercuric chloride was approved by the Scripps Research Institute Department of Environmental Health and Safety.

### 2.7. Statistics

Unless otherwise noted, all data is expressed as mean and standard error. Statistical analysis was done using GraphPad Software, San Diego, CA. Mann-Whitney *U* test was used for comparisons between individual mouse strains. Analysis of variance (ANOVA) with Bonferroni's Multiple Comparison test was used for comparisons between features of mHgIA in (SJL/JxDBA/2)F2 intercross mice. *P* < 0.05 was considered significant.

## 3. Results

### 3.1. *Daf1* mRNA Expression is Reduced in Autoimmune Prone Mice

**Figure 1:**
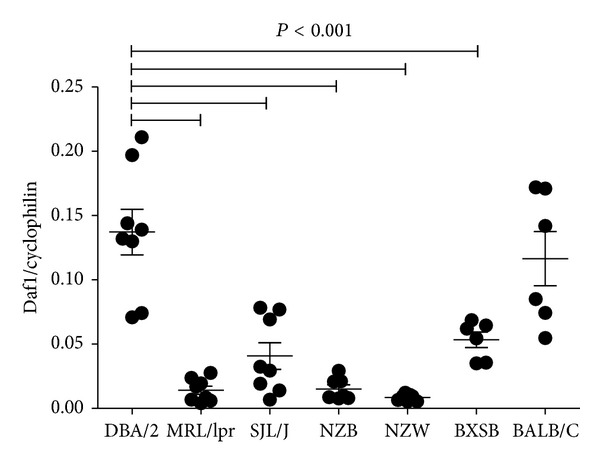
*Daf1* mRNA is reduced in autoimmune prone mice. Real time PCR was used to determine* Daf1* mRNA in spleen cells of naïve female autoimmune prone MRL-*Fas* ^*lpr*^ (MRL/lpr), NZB, NZW, and BXSB mice and healthy DBA/2 and BALB/c mice.* Daf1* was expressed relative to cyclophilin A. *N* = 4-5 mice/strain.

In a previous study, we found that autoimmune prone NZB mice have reduced endogenous DAF1 expression [24]. To determine if this is common to other lupus-prone strains we analyzed* Daf1* mRNA expression in spleen cells from naïve female autoimmune prone MRL-*Fas* ^*lpr*^ (MRL/lpr), NZB, NZW, and BXSB mice and healthy DBA/2 and BALB/c mice (Figure 1). All the autoimmune prone strains had lower* Daf1* mRNA levels than the DBA/2 confirming that reduced DAF1 expression is coupled to a predisposition for autoimmunity.* Daf1* expression in BALB/c mice was no different from DBA/2 but was higher than the other strains tested (*P* < 0.01). Interestingly, SJL/J mice also had reduced* Daf1* mRNA expression. This may reflect the propensity of SJL/J mice to develop autoimmunity with age [32] but may also be a manifestation of dysferlin deficiency in these mice [33]. Thus,* Daf1* is reduced in strains with a predisposition to autoimmunity.

### 3.2. Intron 2 of* Daf1* Contains a Pentanucleotide Tandem Repeat

**Figure 2:**
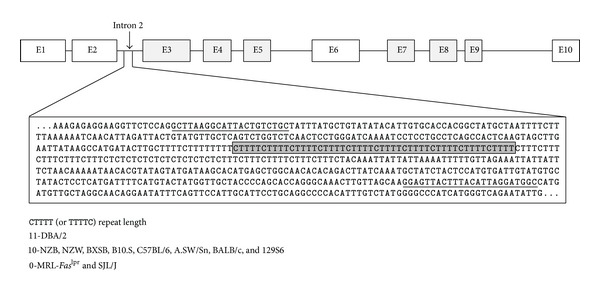
*Daf1* gene organization showing location of the CTTTT_n_ tandem repeat. Diagrammatic representation of exons (E) in* Daf1* (top) and the expanded region of sequence in intron 2 (bottom) showing the CTTTT tandem repeat (shaded box) and PCR primers sequences (underlined). DNA sequence is from the C57BL/6 genomic sequence which has ten CTTTT repeats. Note that the repeat can also be represented by TTTTC by simple removal of the C residue at the 5′ end and inclusion of the C at the 3′ end. DBA/2 mice had the longest repeat sequence with CTTTT (or TTTTC) being repeated 11 times. NZB, NZW, BXSB, B10.S, C57BL/6, A.SW/Sn, BALB/c, and 129S6 mice had 10 repeats while MRL-*Fas* ^*lpr*^ and SJL/J lacked the repeat.

Differences in expression of DAF1 might reflect genetic polymorphisms among the different strains. Sequencing of* Daf1* transcripts and 2.5 kb of genomic DNA 5′ of the* Daf1* ATG start site in NZB and DBA/2 mice revealed no differences compared to the C57BL/6 genome. However, further examination identified a tandem repeat in the second intron of* Daf1* (Figure 2). Sequencing of this region in a number of mouse strains revealed three different genotypes. DBA/2 mice had the longest repeat sequence with CTTTT (or TTTTC) being repeated 11 times. NZB, NZW, BXSB, B10.S, C57BL/6, A.SW/Sn, BALB/c, and 129S6 mice had 10 repeats while MRL-*Fas* ^*lpr*^ and SJL/J lacked the tandem repeat. Tandem repeat length did not show a strict correlation with* Daf1* mRNA expression, although the longest repeat was found in the strain with the highest expression (DBA/2) while the two strains lacking the tandem repeat, MRL-*Fas* ^*lpr*^ and SJL/J, did have significantly lower expression than the DBA/2 (Figure 1). The inability of mercury exposure to decrease DAF1 expression in mHgIA resistant DBA/2 mice [24] suggested the possibility that the DBA/2 tandem repeat may influence the expression of* Daf1* and, in turn, the severity of autoimmunity.

### 3.3. Presence of the DBA/2* Daf1* Tandem Repeat Is Associated with Reduced Immune Deposits

**Figure 3:**
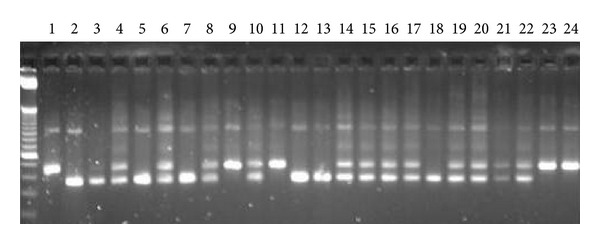
PCR determination of presence or absence of CTTTT tandem repeat. Archived DNA from mercury treated (SJL/JxDBA/2)F2 intercross mice was subjected to PCR using appropriate primers (see Figure 2) and products separated by agarose gel electrophoresis. Lane 1, base pair marker; lane 2, homozygous D tandem repeat; lanes 3 and 4, homozygous S tandem repeat; lane 5, heterozygous D/S tandem repeat; lanes 6–25, (SJL/JxDBA/2)F2.

To determine if the DBA/2 tandem repeat variant is associated with facets of mHgIA we examined archived DNA samples from 133 mice from a (SJL/JxDBA/2)F2 intercross which had been used to identify the* Hmr1* locus [31]. PCR analysis determined that there were 32 mice with the DBA/2 (D/D) tandem repeat: 28 had the SJL/J (S/S) genotype as they lacked the tandem repeat and 73 were heterozygous (D/S) animals (Figure 3). Tandem repeat status was then compared to previously obtained data [31] of immune deposits in kidney and spleen and serum autoantibodies to determine the relationship between presence or absence of the tandem repeat and severity of mHgIA.

**Figure 4:**
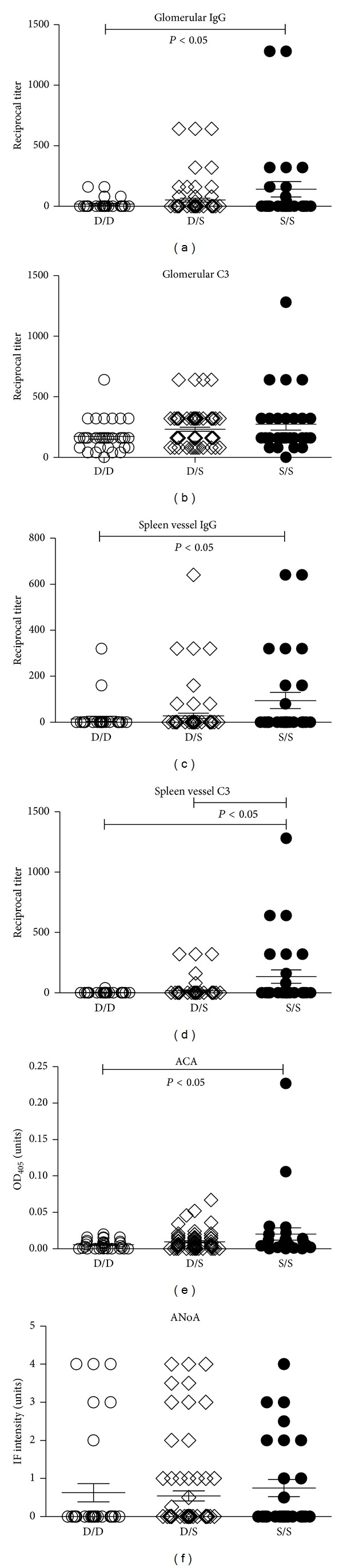
Presence of the DBA/2 tandem repeat is associated with reduced immune deposits. Tandem repeat genotypes (D/D, S/S D/S) were compared with features of mHgIA including glomerular deposits of IgG and C3, splenic vessel deposits of IgG and C3, antichromatin autoantibodies (ACA), and antinucleolar autoantibodies (ANoA) in 133 (SJL/JxDBA/2)F2 intercross mice.

Comparison of autoantibody responses revealed that D/D, S/S, and D/S tandem repeat groups showed no differences in antinucleolar autoantibody (ANoA) response but the antichromatin autoantibody (ACA) response was greater in S/S than D/D animals (*P* < 0.05) (Figure 4). Comparison of immune deposits found that D/D animals had reduced glomerular IgG deposits (*P* < 0.05) compared to S/S animals but no differences were found for C3 deposits (Figure 4) or glomerular IgM. Immune deposits in the spleen were also affected by the presence of the D/D tandem repeat with D/D animals having reduced IgG and C3 deposits in splenic vessels compared to S/S animals (*P* < 0.05) (Figure 4). Splenic vessel deposits of C3 were also reduced in D/S animals compared to S/S animals (*P* < 0.05). Of the 133 animals, 13 had IgG deposits in both kidney glomeruli and splenic vessels and of these 6 (46%) were S/S, 5 (38%) D/S, and 2 (15%) D/D. When expressed as a percentage of each genotype this revealed that 21% of the S/S mice had deposits in both organs, while only 7% of D/S and 6% of D/D had such deposits. None of 8 DBA/2 mice had deposits in either organ while 5/8 SJL/J had deposits with 4 of these having deposits in kidney and spleen. These observations suggest that heterozygous and particularly homozygous presence of the D tandem repeat of* Daf1* is associated with less severe disease.

### 3.4. MHC Class II Genotype Is Not Associated with Reduced Immune Deposits

**Figure 5:**
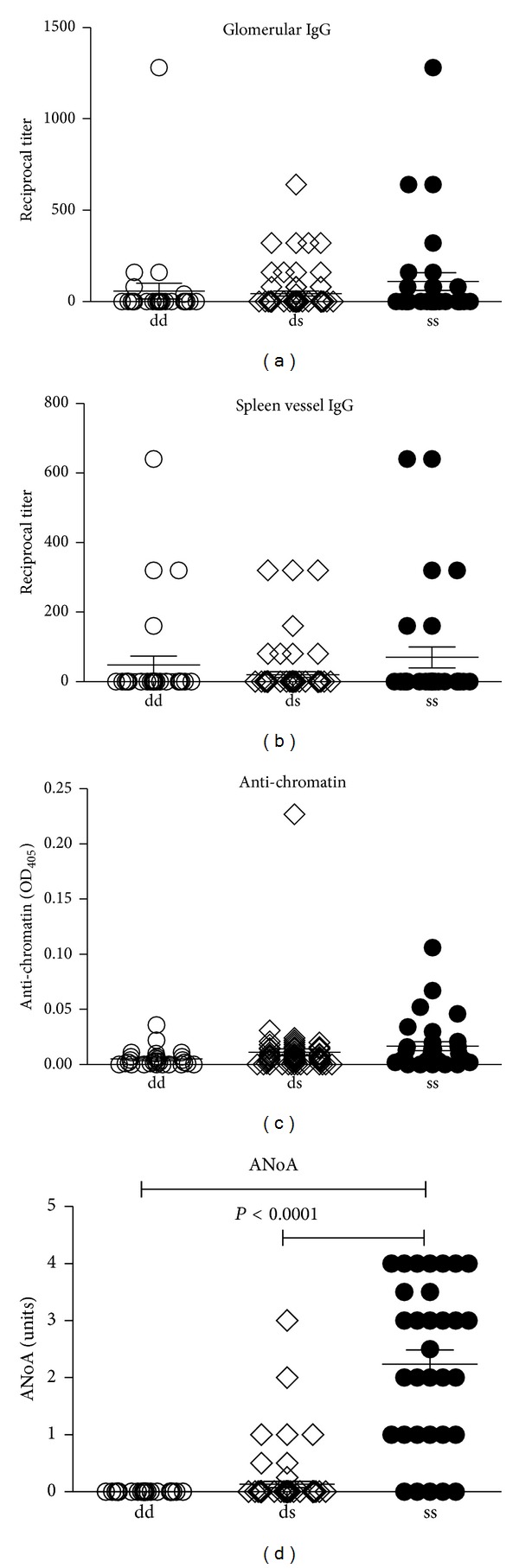
MHC class II is not associated with reduced immune deposits. MHC class II genotypes of (SJL/JxDBA/2)F2 intercross mice (H-2^d^, dd; H-2^s^, ss H-2^ds^, ds) were compared with features of mHgIA including glomerular deposits of IgG, splenic vessel deposits of IgG, antichromatin autoantibodies (ACA), and antinucleolar autoantibodies (ANoA). *N* = 133.

Autoimmunity, including mHgIA [31], is associated with class II genes of the MHC [34]. Therefore, differences in severity of mHgIA in the (SJL/JxDBA/2)F2 intercross could simply reflect distribution of DBA/2 and SJL/J MHC class II genes. To examine this possibility comparison was made between the MHC class II genotypes and the presence of immune deposits in kidney and spleen and serum autoantibodies. MHC class II was not associated deposits of IgG (Figure 5) or C3. Antichromatin autoantibodies were also not associated with MHC class II but, as described previously [31, 35], ANoA was highly associated with mice that were homozygous for H-2^s^ (*P* < 0.0001) (Figure 5). Therefore immune deposits do not reflect of the distribution of DBA/2 or SJL/J MHC.

### 3.5. Expression of* Daf1* Is Not Regulated within the* Hmr1* Locus

**Figure 6:**
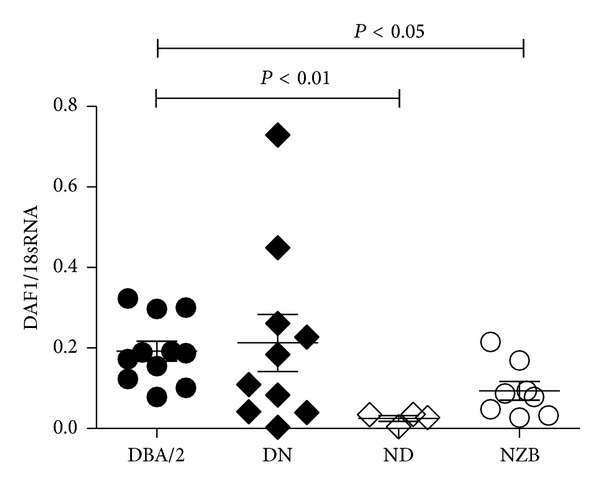
*Hmr1* locus does not control* Daf1* expression. NZB and DBA/2 mice were made congenic for the* Hmr1* locus and* Daf1* expression determined from spleen cells. NZB.DBA/2-*Hmr*1(*Daf*1^DBA/2^) = ND; DBA/2.NZB-*Hmr*1(*Daf*1^NZB^) = DN; *N* = 4–11.

Strain specific differences in the expression of* Daf1* [24] and the relationship of* Hmr1* to disease severity [31] suggested that transfer of the DBA/2* Hmr1* locus into autoimmune susceptible mice may help determine if* Daf1* expression was controlled within the* Hmr1* locus. NZB mice made congenic for the DBA/2* Hmr1* locus (NZB.DBA/2-*Hmr*1(*Daf*1^DBA/2^)  or  ND) still had reduced* Daf1* expression, while DBA/2 mice congenic for the NZB locus (DBA/2.NZB-*Hmr*1(*Daf*1^NZB^)  or  DN) retained the elevated expression of* Daf1* (Figure 6). Thus,* Daf1* expression is affected by genetic elements outside of the* Hmr1* locus.

### 3.6. *Daf1* Transcription Factor Expression Is Not Reduced in Autoimmune Prone Mice

**Figure 7:**
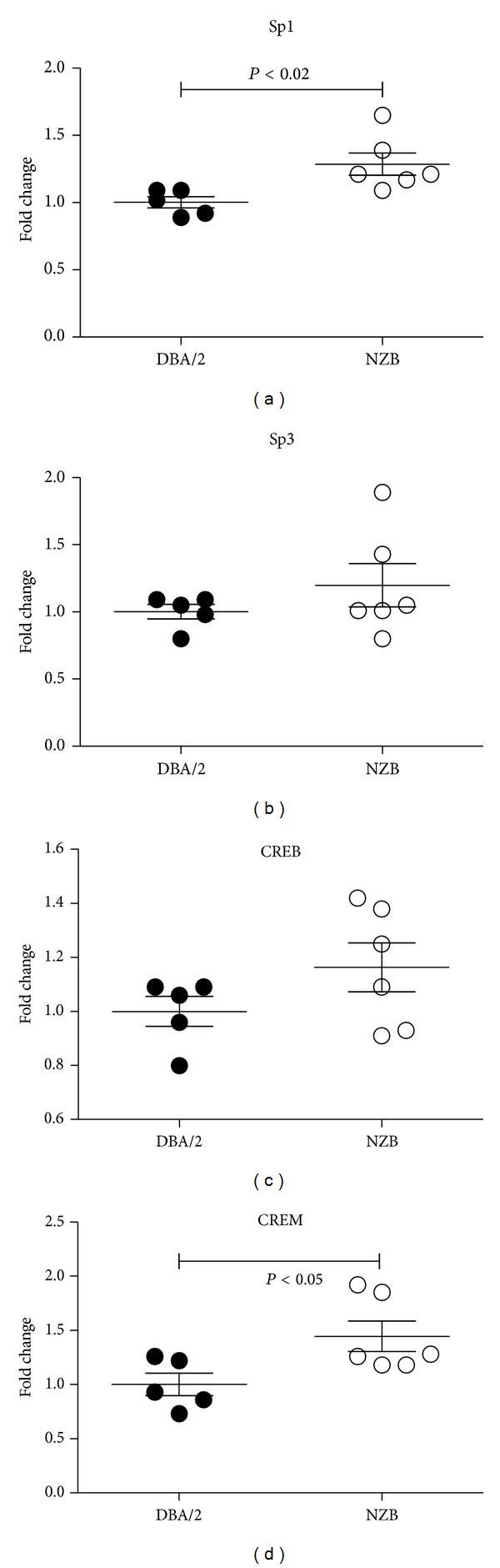
*Daf1* transcription factor expression is not reduced in autoimmune prone mice Real time PCR was used to determine* Sp1, Sp3, CREB, *and* CREM* mRNA in spleen cells of naïve female autoimmune prone NZB and healthy DBA/2 mice. mRNA levels were determined by the ΔΔCT method and normalized against cyclophilin A. Data are expressed as fold change compared to mRNA levels measured in DBA/2 samples. *N* = 5 DBA/2 and 6 NZB.

We previously determined that constitutive expression of* Daf1* is under the control of the transcription factor SP1 [22] which suggested that the reduced expression of* Daf1* in autoimmune prone mice may be due to reduced expression of* Sp1*. Real time PCR analysis of* Sp1* in splenocytes revealed increased expression in NZB compared to DBA/2 mice although Sp3, another member of the Sp1 family, showed no difference in expression (Figure 7). The* Daf1* promoter also contains a CREB binding site [22], however expression of this transcription factor was not different between the two mouse strains although the closely related CREM was increased in NZB mice (Figure 7). Thus reduced* Daf1* in NZB mice could not be attributed to the lack of putative transcription factors, including SP1.

## 4. Discussion

In this study, we extended our observation that lupus-prone NZB mice have reduced DAF1 [24] to other major lupus strains including MRL-*Fas* ^*lpr*^, NZW, and BXSB mice. Thus, reduction of DAF1 is closely associated with susceptibility to autoimmunity. Examination of the* Daf1* sequence revealed a pentanucleotide tandem repeat of either (CTTTT)_n_ or (TTTTC)_n_ in intron 2, with DBA/2 mice having the most repeats while most other strains had one fewer repeat except for MRL-*Fas* ^*lpr*^ and SJL/J mice, which completely lacked the repeat. These observations suggested that resistance of DBA/2 to mercury-induced DAF1 downregulation and subsequent mHgIA might be related to the presence of the longer tandem repeat. Comparison of the presence and absence of the tandem repeat with features of mHgIA supported this possibility by revealing that absence of the repeat was linked with more severe disease particularly IgG deposits. However, mice congenic for the* Hmr1* locus [31], which contains* Daf1*, demonstrated that presence of the DBA/2 tandem repeat in NZB mice and vice versa did not influence* Daf1* expression. Furthermore,* Daf1* expression was not related to an increase in the transcription factor SP1 which has been shown to regulate constitutive expression of* Daf1* [22].

Several lines of evidence have previously suggested that* Daf1* is the most likely gene within the* Hmr1* to explain the association with glomerular immune complex deposits. First, gene expression profiling of NZB and DBA/2 mice exposed to mercury identified 12 differentially expressed genes within the* Hmr1* including* Daf1* [36]. As expected,* Daf1* had greater expression in mHgIA-resistant DBA/2 mice relative to the autoimmune-prone NZB. Moreover,* Daf1* was the only gene with a functional activity, inhibition of complement activation, which offers an explanation for the phenotype displayed by the* Hmr1* locus. Thus the biological role of DAF1 as a negative regulator of complement activation points to its association with deposition of immune complexes. Second, the DBA/2 mouse does not develop mHgIA [31, 37] and Hg exposure does not affect its expression of DAF1 [24]. Third, the SJL/J, which lacks the tandem repeat, has significantly reduced* Daf1* [33], is highly susceptible to mHgIA, and develops significant immune deposits [37]. Our finding that mice congenic for the* Hmr1* do not display any difference in* Daf1* expression compared to their original strain suggests that the association of the absence of* Daf1* tandem repeat with immune deposits might simply reflect the presence of lupus-predisposing genetic variants within the* Hmr1* locus [31]. However, it is also possible that the* Daf1*-expression mediated by the tandem repeat is in epistasis with other genetic variants present in lupus mice.

Our previous studies showed that constitutive expression of* Daf1* is regulated by the transcription factor SP1 [22], but we were unable to demonstrate a correlation of constitutive* Sp1* expression with DAF1 levels. Thus, the reduced expression of DAF1 in autoimmune-prone mice likely involves multiple factors. One intriguing possibility is that the TTTTC pentanucleotide sequence may contribute to* Daf1* expression as it has been identified as an IFN-*γ* and IRF1 response element of the mouse RANTES promoter [38], both IFN-*γ* and IRF1 are required for mHgIA [39], and deficiency of* Daf1* is associated with increased IFN-*γ* [4]. It can be speculated that the lack the pentanucleotide repeat in lupus-prone MRL-*Fas* ^*lpr*^ and the mHgIA-sensitive SJL/J might reduce IRF1s influence on* Daf1* transcription. This is supported by the observation that mice deficient in IFN-*γ* or IRF1 have reduced DAF1 on activated CD4^+^ T cells following mercury exposure even though they do not develop mHgIA [39]. Other mice that are sensitive to mHgIA, such as the B10.S, do have the tandem repeat, and naïve mice have approximately equivalent DAF1 on CD4^+^ T cells as DBA/2 mice [24]. However, induction of mHgIA in the B10.S results in a reduction of DAF1 to levels found in naive NZB while DBA/2 are unaffected [24]. This suggests the possibility that the presence of an additional cis-acting element in the DBA/2 is even more efficient at maintaining* Daf1* expression.

Although the role of the tandem repeat sequence, (CTTTT)n or (TTTTC)n, in* Daf1* expression and mHgIA remains to be resolved, similar sequences in other genes have been shown to influence biological responses. CTTTT or TTTTC repeats have been found in human HLA [40], the CD4 locus [41], and the murine RANTES promoter [38]. The repeat at the CD4 locus has been associated with type I diabetes [42] and vitiligo [43]. A similar sequence, (CCTTT)n, has been found in the promoter of human inducible nitric oxide synthase (NOS2A) where 14 repeats are associated with absence of diabetic retinopathy [44]. IL-1*β* induction of NOS2A with 9, 12, or 15 repeats was inhibited by the presence of 25 mM glucose while the 14 repeats maintained transcription. Whether the size of the tandem repeat influences* Daf1* promoter activity in idiopathic and/or mercury-induced autoimmunity remains to be determined.

## 5. Conclusions

These studies show that expression of decay accelerating factor 1 is reduced in mice susceptible to systemic autoimmunity. Control of* Daf1* expression appears to be multifactorial and is not primarily mediated by a pentanucleotide tandem repeat in intron 2 nor constitutive differences in SP1 expression. The absence of the tandem repeat was associated with increased severity of immune deposits in mercury-induced autoimmunity, which may be due to linkage of the repeat with other predisposing variants or epistasis with other genes that promote* Daf1* expression.

## References

[1] Lublin DM, Atkinson JP (1989). Decay-accelerating factor: biochemistry, molecular biology, and function. *Annual Review of Immunology*.

[2] Song W-C (2004). Membrane complement regulatory proteins in autoimmune and inflammatory tissue injury. *Current Directions in Autoimmunity*.

[3] Heeger PS, Lalli PN, Lin F (2005). Decay-accelerating factor modulates induction of T cell immunity. *Journal of Experimental Medicine*.

[4] Liu J, Miwa T, Hilliard B (2005). The complement inhibitory protein DAF (CD55) suppresses T cell immunity in vivo. *Journal of Experimental Medicine*.

[5] Longhi MP, Harris CL, Morgan BP, Gallimore A (2006). Holding T cells in check—a new role for complement regulators?. *Trends in Immunology*.

[6] Song W-C (2006). Complement regulatory proteins and autoimmunity. *Autoimmunity*.

[7] Post TW, Arce MA, Liszewski MK, Thompson ES, Atkinson JP, Lublin DM (1990). Structure of the gene for human complement protein decay accelerating factor. *Journal of Immunology*.

[8] Spicer AP, Seldin MF, Gendler SJ (1995). Molecular cloning and chromosomal localization of the mouse decay-accelerating factor genes: duplicated genes encode glycosylphosphatidylinositol-anchored and transmembrane forms. *Journal of Immunology*.

[9] Lin F, Fukuoka Y, Spicer A (2001). Tissue distribution of products of the mouse decay-accelerating factor (DAF) genes. Exploitation of a Daf1 knock-out mouse and site-specific monoclonal antibodies. *Immunology*.

[10] Thomas DJ, Lublin DM (1993). Identification of 5′-flanking regions affecting the expression of the human decay accelerating factor gene and their role in tissue-specific expression. *Journal of Immunology*.

[11] Nicholson-Weller A, March JP, Rosen CE (1985). Surface membrane expression by human blood leukocytes and platelets of decay-accelerating factor, a regulatory protein of the complement system. *Blood*.

[12] Medof ME, Walter EI, Rutgers JL (1987). Identification of the complement decay-accelerating factor (DAF) on epithelium and glandular cells and in body fluids. *Journal of Experimental Medicine*.

[13] Spiller OB, Criado-Garcia O, Rodriguez de Cordoba S, Morgan BP (2000). Cytokine-mediated up-regulation of CD55 and CD59 protects human hepatoma cells from complement attack. *Clinical and Experimental Immunology*.

[14] Cocuzzi ET, Bardenstyein D, Stavitsky A, Sudarraj N, Medof ME (2001). Upregulation of DAF (CD55) on orbital fibroblasts by cytokines. Differential effects of TNF-*β* and TNF-*α*. *Current Eye Research*.

[15] Ahmad SR, Lidington EA, Ohta R (2003). Decay-accelerating factor induction by tumour necrosis factor-*α*, through a phosphatidylinositol-3 kinase and protein kinase C-dependent pathway, protects murine vascular endothelial cells against complement deposition. *Immunology*.

[16] Holla VR, Wang D, Brown JR, Mann JR, Katkuri S, DuBois RN (2005). Prostaglandin E2 regulates the complement inhibitor CD55/decay-accelerating factor in colorectal cancer. *Journal of Biological Chemistry*.

[17] Andoh A, Kinoshita K, Rosenberg I, Podolsky DK (2001). Intestinal trefoil factor induces decay-accelerating factor expression and enhances the protective activities against complement activation in intestinal epithelial cells. *Journal of Immunology*.

[18] Saklatvala J, Dean J, Clark A (2003). Control of the expression of inflammatory response genes. *Biochemical Society Symposium*.

[19] Kendall G, Crankson H, Ensor E (1996). Activation of the gene encoding decay accelerating factor following nerve growth factor treatment of sensory neurons is mediated by promoter sequences within 206 bases of the transcriptional start site. *Journal of Neuroscience Research*.

[20] Louis NA, Hamilton KE, Kong T, Colgan SP (2005). HIF-dependent induction of apical CD55 coordinates epithelial clearance of neutrophils. *FASEB Journal*.

[21] Ewulonu UK, Ravi L, Medof ME (1991). Characterization of the decay-accelerating factor gene promoter region. *Proceedings of the National Academy of Sciences of the United States of America*.

[22] Cauvi DM, Cauvi G, Pollard KM (2006). Constitutive expression of murine decay-accelerating factor 1 is controlled by the transcription factor Sp1. *Journal of Immunology*.

[23] Kim DD, Song WC (2006). Membrane complement regulatory proteins. *Clinical Immunology*.

[24] Cauvi DM, Cauvi G, Pollard KM (2007). Reduced expression of decay-accelerating factor 1 on CD4^+^ T cells in murine systemic autoimmune disease. *Arthritis and Rheumatism*.

[25] Miwa T, Maldonado MA, Zhou L (2002). Deletion of decay-accelerating factor (CD55) exacerbates autoimmune disease development in MRL/lpr mice. *American Journal of Pathology*.

[26] Toomey CB, Cauvi DM, Song W-C, Pollard KM (2010). Decay-accelerating factor 1 (Daf1) deficiency exacerbates xenobiotic-induced autoimmunity. *Immunology*.

[27] Cauvi DM, Toomey CB, Pollard KM (2012). Depletion of complement does not impact initiation of xenobiotic-induced autoimmune disease. *Immunology*.

[28] Sogabe H, Nangaku M, Ishibashi Y (2001). Increased susceptibility of decay-accelerating factor deficient mice to anti-glomerular basement membrane glomerulonephritis. *Journal of Immunology*.

[29] Lin F, Salant DJ, Meyerson H, Emancipator S, Morgan BP, Medof ME (2004). Respective roles of decay-accelerating factor and CD59 in circumventing glomerular injury in acute nephrotoxic serum nephritis. *Journal of Immunology*.

[30] Lin F, Kaminski HJ, Conti-Fine BM, Wang W, Richmonds C, Edward Medof M (2002). Markedly enhanced susceptibility to experimental autoimmune myasthenia gravis in the absence of decay-accelerating factor protection. *Journal of Clinical Investigation*.

[31] Kono DH, Park MS, Szydlik A (2001). Resistance to xenobiotic-induced autoimmunity maps to chromosome 1. *Journal of Immunology*.

[32] Owens MH, Bonavida B (1976). Immune functions characteristic of SJL/J mice and their association with age and spontaneous reticulum cell sarcoma. *Cancer Research*.

[33] Kobayashi K, Izawa T, Kuwamura M, Yamate J (2011). Comparative gene expression analysis in the skeletal muscles of dysferlin-defcient SJL/J and A/J mice. *Journal of Toxicologic Pathology*.

[34] Relle M, Schwarting A (2012). Role of MHC-linked susceptibility genes in the pathogenesis of human and murine lupus. *Clinical and Developmental Immunology*.

[35] Hultman P, Bell LJ, Enestrom S, Pollard KM (1993). Murine susceptibility to mercury. II. Autoantibody profiles and renal immune deposits in hybrid, backcross, and H-2d congenic mice. *Clinical Immunology and Immunopathology*.

[36] Cauvi DM, Hultman P, Pollard KM, McQueen CA (2010). Autoimmune models. *Comprehensive Toxicology*.

[37] Hultman P, Bell LJ, Enestrom S, Pollard KM (1992). Murine susceptibility to mercury. I. Autoantibody profiles and systemic immune deposits in inbred, congenic, and intra-H-2 recombinant strains. *Clinical Immunology and Immunopathology*.

[38] Liu J, Guan X, Ma X (2005). Interferon regulatory factor 1 is an essential and direct transcriptional activator for interferon *γ*-induced RANTES/CC15 expression in macrophages. *Journal of Biological Chemistry*.

[39] Pollard KM, Hultman P, Toomey CB (2012). Definition of IFN-*γ*-related pathways critical for chemically-induced systemic autoimmunity. *Journal of Autoimmunity*.

[40] Vorechovsky I, Kralovicova J, Laycock MD (2001). Short tandem repeat (STR) haplotypes in HLA: an integrated 50-kb STR/linkage disequilibrium/gene map between the RING3 and HLA-B genes and identification of STR haplotype diversification in the class III region. *European Journal of Human Genetics*.

[41] Tishkoff SA, Dietzsch E, Speed W (1996). Global patterns of linkage disequilibrium at the CD4 locus and modern human origins. *Science*.

[42] Kristiansen OP, Zamani M, Johannesen J (1998). Linkage and association between a CD4 gene polymorphism and IDDM in Danish IDDM patients. *Diabetes*.

[43] Zamani M, Tabatabaiefar MA, Mosayyebi S, Mashaghi A, Mansouri P (2010). Possible association of the CD4 gene polymorphism with vitiligo in an Iranian population: experimental dermatology. *Clinical and Experimental Dermatology*.

[44] Warpeha KM, Xu W, Liu L (1999). Genotyping and functional analysis of a polymorphic (CCTTT)(n) repeat of NOS_2_A in diabetic retinopathy. *FASEB Journal*.

